# Patient-centred care for cardiovascular risk patients: insights into goal attainment and satisfaction from the DECADE study

**DOI:** 10.1186/s12872-026-05953-z

**Published:** 2026-05-18

**Authors:** Willy Gräfe, Iris Tinsel, Maja Börger, Thomas Kloppe, Andy Maun, Henna Riemenschneider

**Affiliations:** 1 Department of General Practice, Faculty of Medicine Carl Gustav Carus, Dresden University of Technology, Fetscherstraße 74, Dresden, 01307 Germany; 2 Section of Health Care Research and Rehabilitation Research, Institute of Medical Biometry and Statistics, Medical Center, Faculty of Medicine, University of Freiburg, Freiburg, Germany; 3 Institute of General Practice / Family Medicine, Medical Centre, Faculty of Medicine, University of Freiburg, Freiburg, Germany; 4 Department of General Practice and Primary Care, University Medical Centre Hamburg-Eppendorf, Hamburg, Germany

**Keywords:** Cardiovascular diseases, Patient-centred care, Goal attainment, Prevention, Primary care

## Abstract

**Background:**

Cardiovascular diseases (CVD) are the leading cause of death worldwide, with lifestyle-related risk factors such as unhealthy diet, physical inactivity or smoking playing a crucial role. Patient-centred care, which actively engages patients in setting and achieving their health goals, has become increasingly important in treatment and prevention. The Goal Attainment Scaling (GAS) provides a structured method to assess individual goal attainment and satisfaction. This study examines the frequency, attainment, and satisfaction with health goals among patients with at least one lifestyle-related risk factor for CVD within the DECADE intervention. The primary objective of the DECADE intervention was to improve patients’ self-management to reduce their cardiovascular disease risk. One part of the study involved assessing health goals.

**Methods:**

The cluster-randomised controlled DECADE study consisted of four study arms. The intervention groups (IG) received evidence-based health materials (IG1) and/or patient-centred follow-up consultations (IG2 and IG3). The control group (CG) received at the beginning (t0) of the intervention and after 12 months (t2) a CVD risk assessment like the intervention groups. The analysis sample comprises *n* = 712. Health goals, their attainment (6-point Likert scale, 0 = not achieved at all/worse than before to 5 = more achieved than before), and satisfaction with goal attainment (5-point Likert Scale, 1 = very satisfied – 5 = very dissatisfied) took place after 6 months (t1) and t2. GAS values were analysed using means, medians and group differences using Kruskal-Wallis tests.

**Results:**

The participants reported a median of six health goals at t1 and t2. The most frequently set goals were healthy diet, regular physical activity, and weight reduction. While most patients reported achieving healthy diet and physical activity goals, weight reduction was less frequently attained. Despite largely attaining their goals (median_t1_=2.74 [0.33-5.00]; median_t2_=2.71 [0.00–5.00]), patients reported that they were only partially satisfied with their results (median_t1_=2.80 [1.00–5.00]; median_t2_=2.75 [1.00–5.00]). Patients receiving follow-up consultations (IG2 and IG3) showed significantly higher GAS scores at t2 than those in groups without consultations (CG and IG1) (median_IG2_=2.80; median_IG3_=2.86 vs. median_CG_=2.50; median_IG1_=2.58, *p* = 0.004). No gender- or income-specific differences were found, but regional differences emerged: Patients in Dresden had significantly higher GAS scores and were more satisfied compared to those in Hamburg and Freiburg (*p* < 0.001).

**Conclusion:**

The findings highlight the potential of patient-centred consultations and the promotion of individual health goals in the treatment and in the prevention of CVD. Follow-up consultations positively influence goal attainment, emphasizing the need for structured patient-centred communication. Interestingly achieving health goals did not correspond to satisfaction of the patients.

**Trial registration:**

The DECADE-study is registered in the German Clinical Trials Register (DRKS-ID: DRKS00025401; Trial registration date: 2021/06/21) and in the International Clinical Trials Registry Platform (ICTRP): https://trialsearch.who.int/Trial2.aspx?TrialID=DRKS00025401.

## Background

Cardiovascular diseases (CVD) are the leading cause of death worldwide [1]. According to WHO, around 10,000 people in Europe die every day due to CVD [2]. The causes include modifiable lifestyle behaviours (e.g., alcohol and tobacco consumption, physical inactivity, poor dietary quality) and multifactorial medical conditions, most notably obesity, which is driven by a complex interplay of genetic, physiological, and environmental determinants [3, 4]. The prevention and treatment of CVD therefore require a holistic strategy aimed to reduce these risk factors and encouraging health-promoting behaviour. In Germany, general practitioners (GPs) serve as the initial point of contact for a wide range of health concerns and are key providers of primary healthcare for the population. One of the main tasks of GPs is the prevention of illness and the associated counselling of their patients [5]. GPs consider it their responsibility to advise and support patients in the development and implementation of health-conscious behaviour [6]. They therefore take a central role in tackling the health issue of CVD.

Lifestyle improvement is a key component in the prevention of cardiovascular diseases [3]. GPs can support patients in making lifestyle changes through patient-centred consultation. Patient-centred care – as an alternative to disease-centred care – considers patients’ preferences in their healthcare [7–9]. Both the German DEGAM guideline on cardiovascular risk counseling in primary care [10] and the 2021 ESC guidelines [3] explicitly emphasize the need for an informed discussion about cardiovascular risk and treatment benefits that is tailored to the patient’s individual needs. This approach to shared decision-making requires that primary care physicians not only perform evidence-based risk assessments but also actively incorporate patient preferences, psychosocial factors, and individual life goals into the development of a personalized prevention plan [3, 10] Studies have shown patient-centred care can lead to shorter hospital stays [11], reduced healthcare service usage [12], increased self-management [12, 13] and improvements in clinical parameters, such as HbA1c levels in people with diabetes [13]. One component of patient-centred care is setting realistic health goals [3, 10].

The Goal Attainment Scaling (GAS) is a method and instrument for measuring individual goal achievement and self-monitoring of the process that is increasingly being used in healthcare [14, 15]. It allows patients to define specific goals tailored to their individual needs, assess their achievement and express their satisfaction with the results [16]. This approach is used in various medical fields, including rehabilitation, geriatrics and psychiatry [14]. Setting health goals can have positive effects on behavioural changes, as well as psychosocial aspects such as quality of life [17–19]. A health goal that is relevant to the patient is more likely to be achieved, whereas a goal perceived as (too) demanding by a patient is less likely to be realised [20]. There is evidence that monitoring of goal progress by a GP increases the likelihood of achieving a change in behaviour [21].

Health goals address key issues that require long-term strategies and measures by individuals. They identify possible deficits in various areas, point out individual needs and possibilities for changes and, based on this, develop strategic approaches to improve health [22]. Focusing on these issues can improve patient-centred prevention approaches for cardiovascular disease. These can be achieved by identifying patients’ needs, evaluating the effectiveness of the measures and analysing group-specific differences. Satisfaction and goal achievement serve as key indicators for the success of the interventions. Due to this a Goal Attainment Scaling was used in the DECADE intervention to systematically evaluate goal attainment and satisfaction in patients with cardiovascular risk factors.

The complex intervention DECADE (“Decision aid, action planning, and follow-up support for patients to reduce the 10-year risk of cardiovascular diseases”) was evaluated in a cluster-randomised controlled trial (cRCT). The study was carried out collaboratively by the medical centres of the Universities of Freiburg, Dresden, and Hamburg, in partnership with GPs in their respective regions. The primary objective of the DECADE intervention was to improve patients’ self-management to reduce their cardiovascular disease risk. Detailed information about the cRCT design is available in the published study protocol [23]. The intervention showed small but positive effects on patient activation (PAM13) and various secondary endpoints [24]. This paper includes an explorative analysis of the GAS data and addresses the following questions.

- Which health goals did patients choose most often?
- To what extent have the patients achieved their health goals after 6 and 12 months?
- How satisfied were the patients with the achievement of their health goals after 6 and 12 months?
- What differences in goal attainment and patient satisfaction can be observed according to gender, geographic region, income group, and intervention group?

## Methods

### Recruitment

The Institutes of General Practice/Family Medicine at the Medical Center of the University of Freiburg, the Faculty of Medicine Carl Gustav Carus at Dresden University of Technology, and the University Medical Center Hamburg-Eppendorf recruited GPs within their respective regions. GP practices in were informed about the DECADE study through multiple recruitment channels, including postal and electronic mail, telephone calls, face-to-face and online events, and publications in medical journals.

The GPs, subsequently, should include twelve patients (30 to 75 years old) with at least one lifestyle-related cardiovascular risk factor. These factors could have been: smoking, overweight or obesity with a body mass index > 25 kg/m², lack of exercise, unhealthy diet, high alcohol consumption, unhealthy stress behaviour and/or sleeping disorder. The exclusion criteria were: any acute cardiovascular event, nursing requirements, severe or severe acute illnesses or short life expectancy, severe cognitive impairments, severe mental illnesses, alcohol addiction, severe eating disorders, pregnancy, planned medical rehabilitation or insufficient understanding of the German language (reading, speaking). A consent form had to be signed by patients in order to participate in the study. The ethics committee of the University of Freiburg (vote No.: 21-1078 on 2021/04/15) first approved the study. Detailed information about the recruitment process for GPs and patients can be found in the publication of the main study results. Especially in the e-supplement [24].

### Randomisation

The cluster-randomised controlled DECADE study comprised four study arms. GPs were randomly allocated to one of four study arms before the interventions began. All study participants (also the control group CG) received an individualised calculation of their cardiovascular risk at the start and the end of the study using the “arriba” calculator [25]. Patients in the intervention group (IG) 1 received printed and web-based evidence-based health materials with decision- and actions-aids. Patients in the IG2 took part in 4–5 structured, patient-centred consultations with their GP over the course of 12 months, where, among other things, health goals were discussed. Patients in the IG3 received both the patient-materials and the patient-centred consultations. All GPs received a brief training according to their study arm. CG: application of the “arriba” CVR-calculator, IG1 implementation of the DECADE-patient materials, IG2: structured, patient-centred communication including shared decision-making, motivational interviewing, and 5-cards [26–28]; IG3: brief training of the complete intervention.

### Measuring instruments

Before the start of the DECADE intervention (baseline t0), after 6 months (t1) and after 12 months (t2), the patients completed a multi-page questionnaire regarding their health, health behaviour and sociodemographic. The t0 questionnaire was completed at the general practice, and the study team contacted the patients by mail for the t1 and t2 surveys. At t1 and t2, patients were surveyed regarding their health goals, including whether they had set any at the beginning of the study, the content of these goals, the degree to which they had achieved them and their satisfaction with the outcomes. Patients were able to select their own goals from a list of 12 health goals and to rate their goal attainment (adapted GAS) [29] on a 6-point Likert scale (0 = not attainted at all/worse than before to 5 = more attainted than before) as well as their satisfaction with goal attainment (self-developed GAS satisfaction; 1 = very satisfied to 5 = very dissatisfied). The data on health goals, their achievement, and satisfaction were self-reported. The answers were used to calculate the mean scores for GAS and GAS satisfaction, which consider the number of health goals set by the respondent.

The twelve health goals were: (1) engaging in regular exercise (at least 30 min per day), (2) following a healthy diet, (3) reducing body weight, (4) becoming a non-smoker/reducing number of cigarettes, (5) giving up/reducing alcohol consumption, (6) dealing positive with stress/ensure relaxation, (7) ensuring healthy sleep, (8) starting treatment in case of comorbid mental illness, (9) regular blood pressure measurement at home in case of high blood pressure, (10) regular blood glucose monitoring for people with diabetes, 11. participating in regular doctor’s appointments in the disease management program (DMP), 12. taking medication regularly as prescribed by the doctor. The patient-reported data was collected from November 2021 to March 2024.

### Statistical analysis

The number of the respective health goals was represented by absolute frequencies and analysed for gender-specific differences. Descriptive analysis was performed by means, standard deviation, median and range to describe data that were normally or not normally distributed. The Kolmogorov-Smirnov test was used to assess normality of GAS and GAS Satisfaction. The Kruskal-Wallis test was used to identify differences between groups in the categories of region, income group, and intervention group. The Mann-Whitney U test was used to identify differences between genders. The significance level was set at *p* ≤ 0.05. In this explorative analysis the 6-point GAS Likert scale was dichotomised (0,1,2 = not achieved and 3,4,5 = achieved) to provide a descriptive account of achievement of the respective health goals.

### Utilisation of AI

ChatGPT and DeepL were used to support the language editing of this manuscript.

## Results

### Sample description

Of the originally planned *n* = 103 GPs, *n* = 76 (73.8%) agreed to participate and were enrolled in the study after signing participation agreements and completing baseline questionnaires prior to randomization. By mid-January 2023, the *n* = 76 participating GPs had enrolled *n* = 797 patients, corresponding to an average of 10.49 patients per GP (SD = 3.16). Recruitment was affected by the COVID-19 pandemic, necessitating a recalculation of the required sample size. The calculation was based on the primary endpoint of patient activation, not on the present explorative analysis of health goals. With a revised power of 80% (originally 90%), an assumed dropout rate of 10%, an ICC of 0.1, and the planned effect size of 0.3 (Cohen’s d), the minimum required sample size was determined to be *n* = 717 patients, which was achieved. Patient-level response rates could not be calculated as GP practices did not systematically document the total number of patients approached.

The analysis sample consisted of *n* = 712 patients (female = 53.9%, male = 46.1%) with a mean age of 56.7 (SD = 10.5) years at t0. Further characteristics of the sample for the baseline survey are shown in Table 1.

**Table 1:** Sample description Baseline

Variables	*n* (%)
Gender	
Female Male	384 (53.9) 328 (46.1)
Region	
Freiburg Hamburg Dresden	262 (36.8) 219 (30.8) 231 (32.4)
Monthly net household income	
< 1500€ 1500€-<3000€ 3000€-<4500€ > 4500€ Missings	73 (10.3) 216 (30.3) 153 (21.5) 126 (17.7) 144 (20.2)
Intervention group	
CG IG1 IG2 IG3	181 (25.4) 160 (22.5) 198 (27.8) 173 (24.3)
Diabetics	188 (26.4)
Smoker	157 (22.1)

### Overview of health goals, goal attainment and satisfaction with goal attainment

**Fig. 1:**
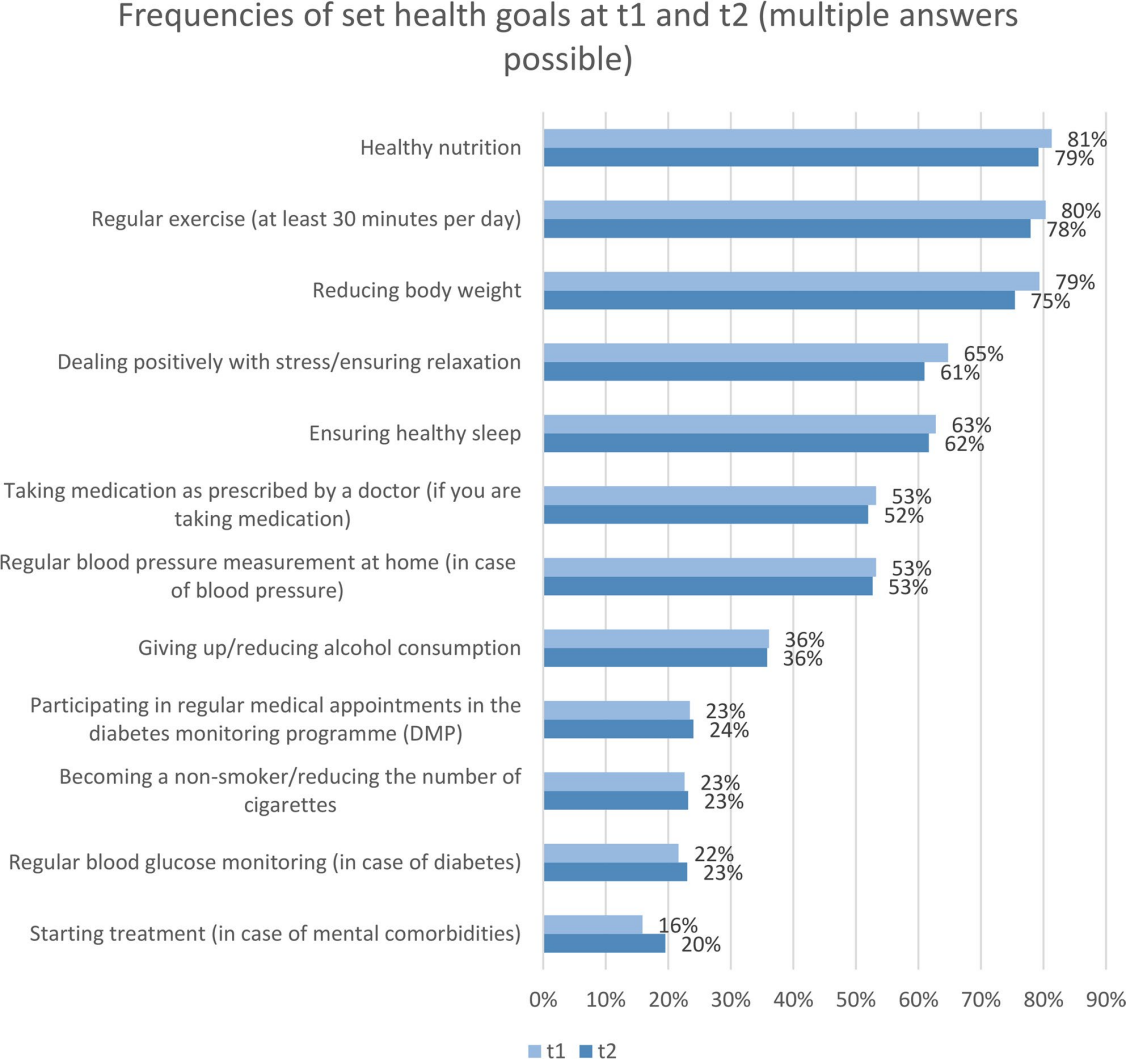
Frequencies of set health goals at t1 (*n* = 680) and t2 (*n* = 639) (multiple answers possible)

The mean number of health goals the patients aimed to achieve at t1 (*n* = 680) was 6.23 (SD = 2.74) and at t2 (*n* = 639) was 6.42 (SD = 2.82; range t1: 1–12; range t2: 0–12). The three most frequently mentioned health goals at t1 and t2 were healthy diet (n_t1_=579 and n_t2_=564), regular exercise (n_t1_=572 and n_t2_=555) and reducing body weight (n_t1_=565 and n_t2_=537) (Fig. 1). There were no gender-specific differences. The median of the GAS at t1 was 2.74 (range: 0.33-5.00), and at t2 2.71 (range: 0.00–5.00). The median GAS satisfaction at t1 was 2.80 (range: 1.00–5.00), and at t2 2.75 (range: 1.00–5.00).

The percentages of health goals achieved are based on the total number of patients who pursued the goal and reported the degree to which they achieved it, i.e., not every patient pursued every health goal (Table 2).

**Table 2:** Number of patients (n) who attempted to achieve health goals at t1 and t2

Health Goals	t1	t2
Engaging in regular exercise (at least 30 min per day)	570	549
Following a healthy diet	577	559
Reducing body weight	559	528
Becoming a non-smoker/reducing number of cigarettes	149	146
Giving up/reducing alcohol consumption	253	250
Dealing positive with stress/ensure relaxation	455	424
Ensuring healthy sleep	443	428
Starting treatment in case of comorbid mental illness	112	133
Regular blood pressure measurement at home in case of high blood pressure	374	366
Regular blood glucose monitoring for people with diabetes	142	143
Participating in regular doctor’s appointments in the disease management program (DMP)	165	164
Taking medication regularly as prescribed by the doctor	378	363

**Fig. 2:**
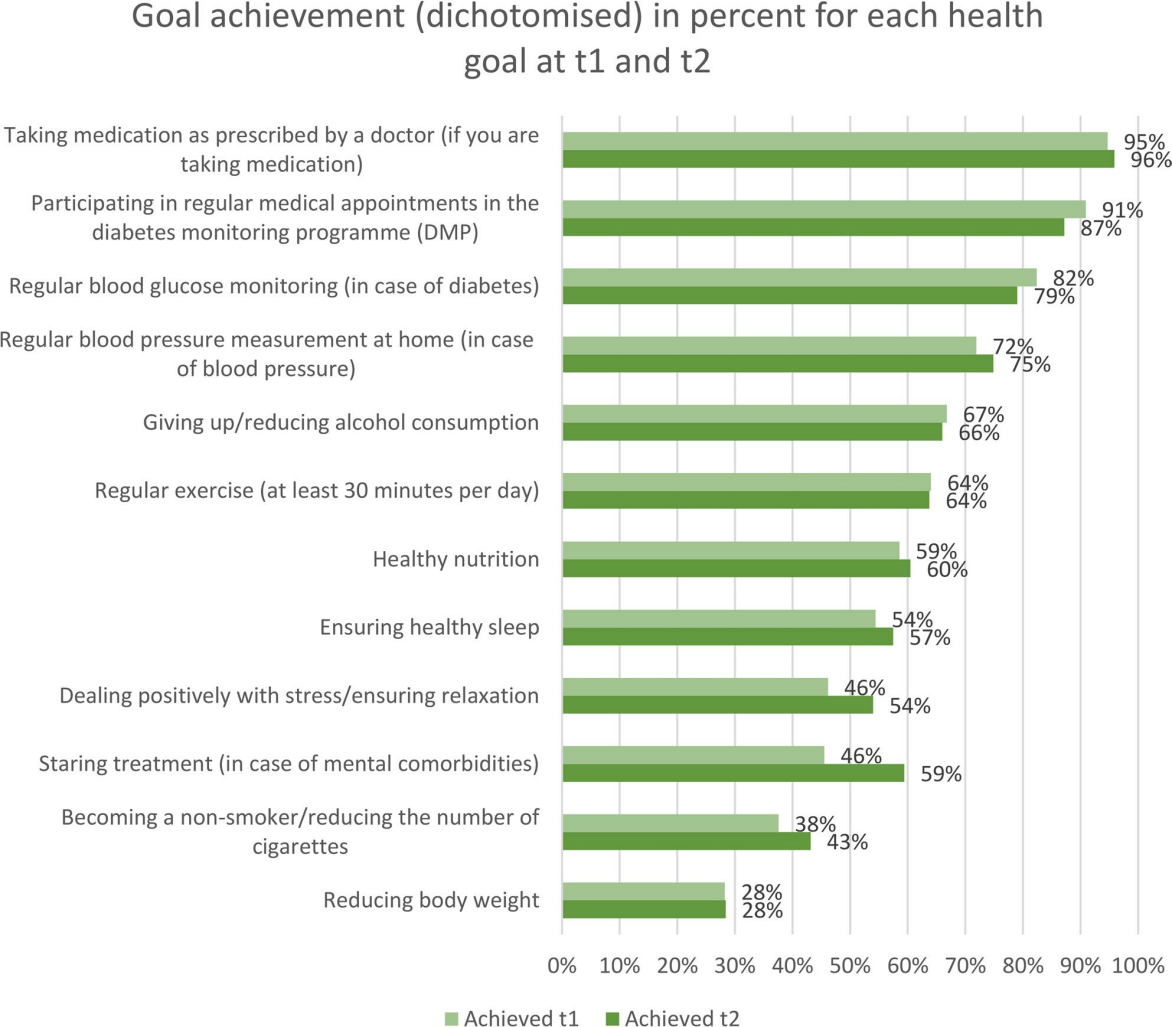
Goal achievement (dichotomised) in percent for each health goal at t1 and t2

**Fig. 3:**
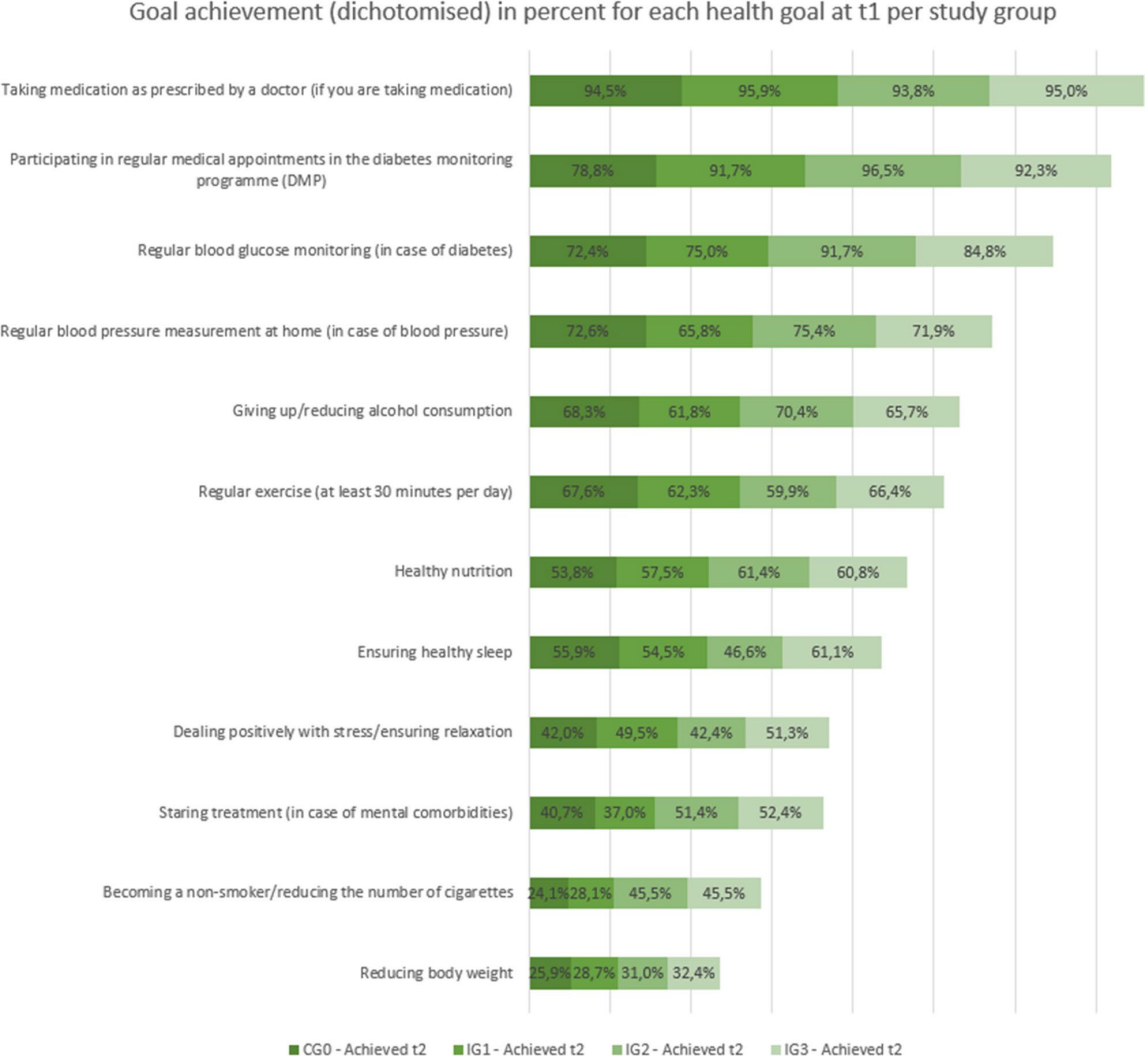
Goal achievement (dichotomised) in percent for each health goal at t1 per study group

**Fig. 4:**
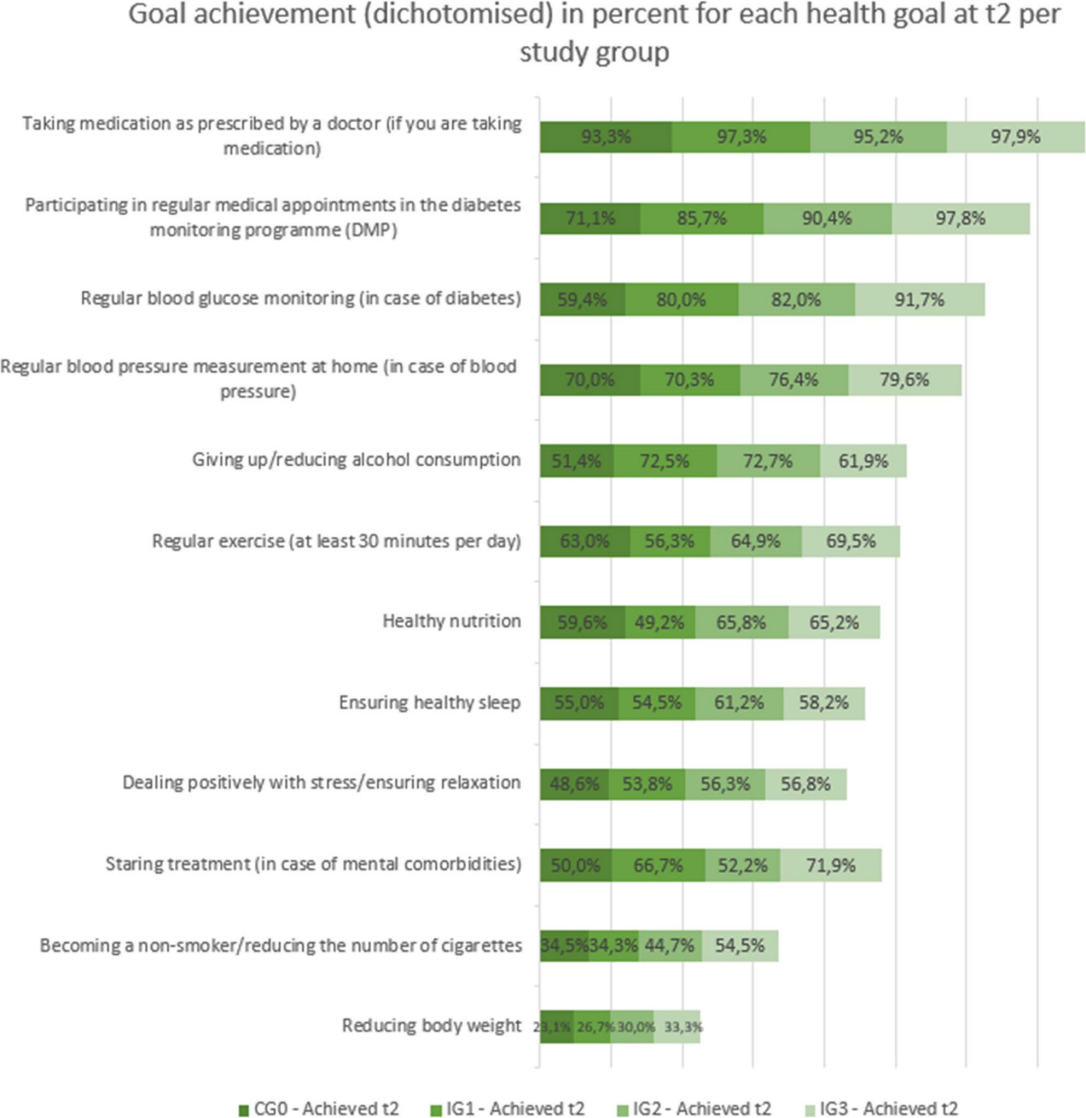
Goal achievement (dichotomised) in percent for each health goal at t2 per study group

The most frequently achieved health goals at t1 and t2 were: taking medication regularly as prescribed by doctor, attending regular appointments in German DMP for diabetes and regular blood glucose monitoring for diabetes. The patients were least likely to achieve the following health goals: Reduce body weight, become a non-smoker/reduce the number of cigarettes and start treatment in the case of comorbid mental illness (Fig. 2). As shown in Figs. 3 and 4, from a descriptive perspective, patients in the groups with additional consultations (IG2 and IG3) achieved their health goals more frequently than patients in the groups without additional consultations (CG0 and IG1) at both measurement time points (t1 and t2).

### Goal attainment and satisfaction with goal attainment by gender, region, income groups and intervention groups

The Kolmogorov-Smirnov test was significant (*p* < 0.001) for both GAS (t1 and t2) and GAS satisfaction (t1 and t2), which is why we treated these variables as non-parametric. The medians of GAS and GAS satisfaction for gender, regions, income groups and intervention groups are shown in Table 3. The study found no gender-specific differences in GAS and GAS satisfaction at both measurement points. Region-specific differences in GAS and GAS satisfaction were identified. Patients in Dresden had higher GAS t1 and GAS t2 values than patients in Freiburg and Hamburg and showed a higher satisfaction with goal attainment than in the other two regions. There were no differences between the income groups in terms of GAS and GAS satisfaction. There were differences in GAS at t2 depending on the intervention group. The intervention groups with the follow-up consultations (IG2 and IG3) had a higher GAS t2 value than the intervention groups without follow-up consultations (CG and IG1) Table 3.

**Table 3:** GAS (t1 and t2) and GAS satisfaction grouped by gender, region, income group and intervention group

Variables	GAS t1 median [range]^1^	GAS t2 median [range]^1^	GAS Satisfaction t1 median [range]^2^	GAS Satisfaction t2 median [range]^2^
Gender				
female male	2.73 [0.33-5.00] 2.75 [0.50-5.00]	2.72 [0.00–5.00] 2.70 [0.17-5.00]	2.79 [1.00–5.00] 2.78 [1.00–5.00]	2.75 [1.00–5.00] 2.75 [1.00-4.88]
Mann-Whitney-U test	Z=-0.60 *p* = 0.55	Z=-0.12 *p* = 0.91	Z=-0.45 *p* = 0.66	Z=-0.31 *p* = 0.76
Region				
Freiburg Hamburg Dresden Kruskall-Wallis test	2.67 [0.33–4.60] 2.60 [0.50-5.00] 2.89 [1.00-4.50] **H = 18.12** *p* < 0.001*	2.67 [0.27-5.00] 2.50 [0.50–4.50] 3.00 [0.00–5.00] **H = 20.61** *p* < 0.001*	2.83 [1.00–5.00] 2.94 [1.00–5.00] 2.67 [1.00–5.00] **H = 14.07** *p* < 0.001*	2.75 [1.00–5.00] 2.88 [1.00–5.00] 2.60 [1.00–5.00] **H = 6.06** *p* < 0.001*
Income group				
< 1500€ 1500-<3000€ 3000-<4500€ > 4500€ Kruskall-Wallis test	2.67 [0.50–4.40] 2.80 [0.33–4.60] 2.67 [0.50-5.00] 2.63 [0.50-5.00] H = 3.613 *p* = 0.306	2.87 [1.14–4.13] 2.78 [0.00-4.42] 2.63 [0.50-5.00] 2.50 [0.50-5.00] H = 7.718 *p* = 0.052	2.74 [1.25-5.00] 2.71 [1.00–5.00] 3.00 [1.00–5.00] 2.86 [1.00–5.00] H = 4.863 *p* = 0.182	2.67 [1.25-5.00] 2.71 [1.00–5.00] 3.00 [1.00–5.00] 2.80 [1.00-4.50] H = 5.866 *p* = 0.118
Intervention group				
CG IG1 IG2 IG3 Kruskall-Wallis test	2.67 [0.33–4.50] 2.69 [0.50-5.00] 2.71 [0.50-5.00] 2.80 [0.50–4.50] H = 5.18 *p* = 0.159	2.50 [0.50–4.50] 2.58 [0.50–4.40] 2.80 [0.17-5.00] 2.86 [0.00–5.00] **H = 13.19** *p* = 0.004*	3.00 [1.00–5.00] 2.80 [1.00–5.00] 2.76 [1.00–5.00] 2.75 [1.00–5.00] H = 2.39 *p* = 0.500	2.80 [1.00–5.00] 3.00 [1.25-5.00] 2.67 [1.00–5.00] 2.67 [1.00–5.00] H = 3.90 *p* = 0.270

*p-value≤ 0.05

^1^GAS: 0 = not achieved at all/worse than before to 5 = more achieved than before

^2^GAS satisfaction: 1 = very satisfied to 5 = very dissatisfied

## Discussion

This exploratory analysis examined the frequencies of goal setting, goal attainment, and satisfaction with goal attainment of patients with at least one lifestyle-related cardiovascular risk factor, within the primary care setting as part of the DECADE intervention. An adapted version of GAS was used to measure goal attainment and satisfaction was measured with self-developed items [23, 24, 29]. GAS has previously been applied to patients with depression, in geriatric care and in rehabilitation setting [14, 20, 30]. However, to the best of our knowledge, GAS has not been used in cardiovascular prevention. Except in the DECADE pilot study [31]. The results of this study show that most patients set lifestyle goals such as healthy diet, regular exercise and weight loss. Among patients who pursued these goals, most reported achieving a healthy diet and regular exercise, but not weight loss. Churilov et al. (2020) showed in their study that goals perceived as difficult are less likely to be achieved [20]. In our study, weight loss seemed to be such a difficult goal for patients to achieve. In addition to behavioural actions, psychological (e.g. self-efficacy expectations) and social (e.g. dietary habits of the social environment) factors are also relevant for sustained weight loss [32]. While the DECADE intervention primarily targeted behavioural factors, the complex interplay with psychological and social determinants should also be considered. In addition, the discrepancy between reported achievement of dietary and exercise goals and the lack of weight loss could be due to that self-reported data on behavioural changes are subject to potential bias, as participants may overestimate their adherence or underestimate their calorie intake. To better understand the discrepancy between reported behavioural changes and clinical outcomes, objective measurement methods (e.g. activity trackers, dietary diaries) would be required in future studies.

The majority of patients stated that they had mostly achieved their health goals. Nevertheless, they were only partially satisfied with the extent of the achievement of their goals. The observed discrepancy between goal attainment and satisfaction may be linked to the discrepancy between current health status and ideal expectations rather than the absolute achievement of pre-defined targets [33]. Research has shown that patient satisfaction is influenced not only by the objective attainment of goals but also by subjective perceptions of progress and the alignment of goals with personal values and priorities [34]. According to Locke and Latham (2002), even when patients have mostly achieved a goal, the perception of it as “not fully achieved” can reduce their satisfaction [35]. An important consideration in interpreting our goal attainment findings is the high mean number of goals set by patients (M_t1_ = 6.2, SD_t1_ = 2.7 and M_t2_ = 6.4, SD_t2_ = 2.8). While goal-setting is a powerful motivational tool, the effectiveness of goals depends critically on their specificity, difficulty, and the cognitive resources available for pursuit [35]. Setting multiple simultaneous goals, particularly in the context of cardiovascular risk reduction where behavioral changes often require substantial effort and lifestyle modification, may lead to cognitive and motivational overload. When patients attempt to address numerous cardiovascular risk factors concurrently—such as diet modification, increased physical activity, stress reduction, and smoking cessation—attentional resources become divided and the likelihood of fully achieving any individual goal may decrease. Future interventions should consider emphasizing goal prioritization, helping patients focus on a smaller, more manageable number of specific goals.

The observed increase in the average number of health goals from t1 to t2 is consistent with recent findings regarding patient empowerment. An increase in goal quantity can be seen as a sign of improved health literacy; as patients better understand their condition through the intervention As patients undergo treatment, their internal conceptualization of “health” often shifts from the mere absence of disease to a holistic state of well-being, leading to the adoption of additional goals [36]. After all, the main aim of the DECADE intervention was to promote patient activation.

The goal attainment of the DECADE intervention appears to be independent of gender and income. Nevertheless, patients in Dresden achieved their health goals more frequently than patients in Hamburg and Freiburg, and they were more satisfied with achieving their goals. This could be due to potential differences in care structures, regional health programmes or cultural factors. Dresden (Saxony), for example, has a more favourable ratio of GPs to residents, which suggests better access to primary care [37]. Despite lower average incomes and a higher risk of poverty in Dresden, these healthcare structures may go some way towards offsetting socio-economic disadvantages [38]. Nevertheless, further studies should investigate these regional differences in greater depth.

The patients in the intervention groups with follow-up consultations (IG2 and IG3) achieved the highest GAS scores. As shown by Harkin et al. (2016) [21] this highlights the importance of structured, patient-centred follow-up contacts for achieving health goals and sustainable self-management of the patients [39]. Descriptively, these patients were also more satisfied with the achievement of their goals. Our finding that structured follow-up consultations (IG2 and IG3) led to significantly higher goal attainment provides empirical support for recommendations in the German DEGAM guideline, which emphasizes the importance of follow-up consultations when lifestyle modifications have been agreed upon [10]. The guideline explicitly recommends renewed counseling when cardiovascular risk factors change or when measures to reduce cardiovascular risk have been implemented. This recommendation reflects the recognition that behavioral change is a process requiring ongoing support rather than a one-time intervention. A principle clearly demonstrated in our results.

However, studies have indicated that GPs often struggle to provide lifestyle counselling due to time constraints or inadequate financial compensation [6, 40]. In the analysis by Graefe et al. (2025), GPs of the DECADE-study stated that interprofessional collaboration in lifestyle counselling could be beneficial [6]. Since the goal of weight loss is difficult to achieve, as shown in our study, collaboration with nutritionists might be very useful or the use of weight-reduction programmes. In the context of primary care, effective patient-centred care necessitates the collaboration of diverse healthcare professionals who engage in ongoing, structured interprofessional communication.

While statistically significant differences in goal attainment emerged between intervention groups, the clinical relevance of these findings is uncertain. Given that GAS was used as a self-reported outcome measure and was not the primary endpoint for sample size calculation, these findings should be interpreted with caution. Future studies are needed to clarify whether training-related variations in physician communication translate into meaningful differences in patient goal-setting and achievement.

### Limitations

Several limitations should be considered when interpreting our findings. First, the recruitment process may have introduced selection bias. GP practices recruited patients during selected consultation time slots through posters, leaflets, and verbal information provided by GPs. As recruitment was not strictly consecutive and GPs played an active role in patient enrollment, it cannot be excluded that some practices preferentially recruited patients with specific cardiovascular risk factors (e.g., smokers) or those perceived as more motivated. To minimize a possible selection bias, GP practices were asked to inform patients during self-designated morning and afternoon consultation time slots. Furthermore, participation in intervention studies typically attracts individuals who are already engaged in health-promoting behaviours. Consequently, our sample may not be fully representative of the general primary care population, and results should be interpreted with caution regarding generalisability to less motivated or health-conscious patients.

One limitation is that the specific changes in general practitioners’ communication patterns could not be directly observed, as such monitoring of implementation would have significantly disrupted routine care and skewed the study results. However, the positive effects on PAM13 changes, participation, and satisfaction with CVR counseling, as well as the improved outcomes in IG2 compared to the pilot study, suggest that the optimized DECADE training had a positive impact on patient-centered CVR counseling.

Due to the exploratory nature of this analysis, no Bonferroni correction was performed. The results are based on self-reports from patients, which can always lead to subjective bias. Goal Attainment Scaling (GAS) was a secondary outcome in the DECADE study. While not a validated instrument, it was adapted specifically for utilisation in this study. Accordingly, the sample size calculation was not based on this outcome. Also, the DECADE study was designed to assess patient activation rather than the comparison of self-reported health goals with objective measurements. Although we collected data on stress, alcohol consumption, diet, and smoking status, these were self-reported, and comparing self-reported data with other self-reported data is not very productive. Future studies could focus specifically on this by comparing patients’ perceptions of their progress toward their goals with objective data.

## Conclusion

The results of this exploratory study suggest that structured patient-centred consultations are beneficial to attain health goals. The implementation of sustainable, interdisciplinary care approaches could further enable long-term improvements in the prevention of cardiovascular diseases. The DECADE intervention showed potential to enhance cardiovascular prevention.

## Data Availability

According to the data sharing plan, the data are not available for third parties. As part of the DECADE study, patients were asked to complete standardized questionnaires, including the licensed PAM13 instrument from Insignia Health, at three time points (t0, t1, t2). According to the original license agreement, limited, anonymized transfer of PAM13 data to Insignia Health was planned. However, no actual data transfer took place because there is no request from Insignia Health and the license agreement has since been terminated. Thus, no third party has gained access to data from the DECADE study.
